# Supplementary material for: Minimal effective dose of bedaquiline administered orally and activity of a long acting formulation of bedaquiline in the murine model of leprosy
Source: PLoS Negl Trop Dis. 2023 Nov 27;17(11):e0011379. doi: 10.1371/journal.pntd.0011379 (PMC10703217; doi:10.1371/journal.pntd.0011379)
Supplement: Attachment — Submitted filename: PNTD_D_23_00593__Response to Reviewers_11.08.23.docx.docx [file pntd.0011379.s001.docx]

**Answers to reviewers**

<b>Key Review Criteria Required for Acceptance?

<b>Methods

-Are the objectives of the study clearly articulated with a clear testable hypothesis stated?

-Is the study design appropriate to address the stated objectives?

-Is the population clearly described and appropriate for the hypothesis being tested?

-Is the sample size sufficient to ensure adequate power to address the hypothesis being tested?

-Were correct statistical analysis used to support conclusions?

-Are there concerns about ethical or regulatory requirements being met?

Reviewer #1: The Objectives and Methods are correctly applied and clearly described. The numbering notation for the bacillary load injected into the mouse-foot-pads is not well displayed in the PDF version that was downloaded, although seems to be correct.

***Answer****: we thank the reviewer for his positive comment.*

Reviewer #2:

C1: The rationale for using immunocompromised nude mice for the first study and immunocompetent mice for the second study could be better explained. Should they be considered as representing different phenotypes on spectrum of leprosy disease? How does the finding of a higher MED in immunocompromised mice compared to prior results in immunocompetent mice impact future development plans? For example, if the ongoing clinical trial evaluating a BDQ dose that is already lower than the current TB dose is successful?

***Answer****: the nude model aims to mimic low-immunity leprosy and the swiss high-immunity leprosy, and therefore multi vs paucibacillary leprosy, respectively. It makes sense that the MED is higher in the nude model. MED measured in both mice models are interesting to choose BDQ dosage in clinical trials. The dose of BDQ to treat patients in the future clinical trials is calculated by taking into account the MED in both immunocompetent mice, immunosuppressed nude mice and the exposure obtained in patients treated with BDQ monotherapy in Brazil (LEP2001 study). This rational has been add into the material and methods section (lines 139-141).*

C2: Could the authors comment on how well the CFZ dose of 20 mg/kg reflects exposures attained with the 50 mg daily dose used in leprosy treatment?

***Answer****: The dosage used in our study is the current dosage used in murine model of leprosy (e.g. Gelber RH, Levy L. Detection of persisting Mycobacterium leprae by inoculation of the neonatally thymectomized rat. Int J Lepr Other Mycobact Dis. 1987 Dec;55(4 Suppl):872-8. PMID: 3325581 ; Ji B, Perani EG, Petinom C, Grosset JH. Bactericidal activities of combinations of new drugs against Mycobacterium leprae in nude mice. Antimicrob Agents Chemother. 1996 Feb;40(2):393-9. doi: 10.1128/AAC.40.2.393. PMID: 8834886; PMCID: PMC163122.); these publications have been added in the manuscript in the material and methods section to justify the dosages used. However, because clofazimine was abandoned as an option for TB treatment shortly after its discovery, and also because the use of clofazimine for leprosy is not based on extensive experimental studies (****Levy L. 1974. Pharmacologic studies of clofazimine. Am J Trop Med Hyg 23:1097–1109****.), the pharmacokinetics (PK) and pharmacodynamics (PD) of the drug are poorly understood, and the dosing for both TB and leprosy patients is not evidence based.* *Very few studies have been done to characterize the PK of clofazimine in humans. A single- and multiple-dose PK studies in uninfected mice suggested that the 25-mg/kg dose administered to the mice resulted in PK properties that were similar to those reported by Williams et al., who administered clofazimine at 20 mg/kg to uninfected BALB/c mice (****Williams K, Minkowski A, Amoabeng O, Peloquin CA, Taylor D, Andries K, Wallis RS, Mdluli KE, Nuermberger EL. 2012. Sterilizing activities of novel combinations lacking first- and second-line drugs in a murine model of tuberculosis. Antimicrob Agents Chemother 56:3114 – 3120. http://dx.doi.org/10.1128/AAC.00384-12****.), and are also similar to what was reported for the 100-mg daily dose in human leprosy patients (****Banerjee DK, Ellard GA, Gammon PT, Waters MF. 1974. Some observations on the pharmacology of clofazimine (B663). Am J Trop Med Hyg 23:1110 –1115****). However, Diacon and colleagues found much lower plasma clofazimine concentrations after 14 days of administration; TB patients who received 300 mg/day of clofazimine for 3 days (i.e., loading doses) followed by 100 mg daily for the following 11 days had a mean plasma drug concentration of 0.2315/ml. Thus, the PK of clofazimine in humans also needs to be better understood. Irwin and colleagues published data from an elegant series of experiments assessing the antimicrobial activity of clofazimine when administered to M. tuberculosis (strain Erdman)-infected BALB/c and C3HeB/FeJ mice (****Irwin SM, Gruppo V, Brooks E, Gilliland J, Scherman M, Reichlen MJ, Leistikow R, Kramnik I, Nuermberger EL, Voskuil MI, Lenaerts AJ. 2014. Limited activity of clofazimine as a single drug in a mouse model of tuberculosis exhibiting caseous necrotic granulomas. Antimicrob Agents Chemother 58:4026 –4034. http://dx.doi.org/10.1128/AAC.02565-14.)****. They found that clofazimine (administered at 20 mg/kg/day) was highly active in BALB/c mice, resulting in reductions of 2.5 and 4.0 log10 CFU in the lungs after 4 and 8 weeks of treatment, respectively. This bactericidal activity is similar to, although slightly more potent than, what was observed another study at all clofazimine doses (****Swanson RV, Adamson J, Moodley C, Ngcobo B, Ammerman NC, Dorasamy A, Moodley S, Mgaga Z, Tapley A, Bester LA, Singh S, Grosset JH, Almeida DV. Pharmacokinetics and pharmacodynamics of clofazimine in a mouse model of tuberculosis. Antimicrob Agents Chemother. 2015;59(6):3042-51. doi: 10.1128/AAC.00260-15. Epub 2015 Mar 9. PMID: 25753644; PMCID: PMC4432183.****). Given these data 20 mg/kg could reflect exposures attained with the 50 mg daily dose used in leprosy treatment, but might also reflect an exposure as high as 100 mg.*

C3: For the statistical analysis of the first experiment, please clarify if the results from mice with AFB below the limit of detection were included and, if so, what value was assigned to them. Zero cannot be log-transformed and a value closer to the lower limit of detection would be more conservative and, arguably, more appropriate for statistical comparisons.

***Answer****: we considered a threshold of log10 4.24 when all mice footpads where negative but zero for the negative mice footpads when there was a mixture of positive and negative mice footpads. However, reviewer 2 is right that is not an appropriate way to present the results. We therefore propose to replace the column representing the mean by a column where only the range of AFB in the positive mouse footpad is give in the revised version of the manuscript. Statistical analysis has been redone by using a Fisher exact teste based on the number of positive and negative mice in the treatment groups compared to the untreated control group.*

C4: The methods should describe how the MED is defined.

***Answer****:* The MED is defined as the lowest dose of a drug that inhibits the growth of *M. leprae,* i.e. corresponding to the group where all the mice footpads remain negative.

Reviewer #3: The methods meet the criteria for acceptance. It is very well written, clear and provides appropriate explanation citing key references across mouse foot pad and relevant drug sensitivity techniques.

***Answer****: we thank the reviewer for his positive comment.*

--------------------

<b>Results

-Does the analysis presented match the analysis plan?

-Are the results clearly and completely presented?

-Are the figures (Tables, Images) of sufficient quality for clarity?

Reviewer #1: Clear and accurate.

***Answer****: we thank the reviewer for his positive comment.*

Reviewer #2:

C1: If there is any information on the PK of these BDQ-LA doses used in the experiment, it should be included to better understand why the 20 mg/kg was less effective when administered as a LA injection rather than orally.

***Answer****: there was no PK data generated with the BDQ-LA 20 mg/kg dose used in this study at the time this study was designed, and the initial goal was to compare both molecules at the same dosage. However, a PK study was performed in Swiss mice that received a single dose of 160 mg/kg by intramuscular injection with data collected at 1, 4, 7, 24, 168, 336, 504, 672, 840, 1,176, 1,512, 1,848, and 2,184 h after injection, after the initiation of our study. The plasma concentrations of BDQ are shown below (more details can be found in Kaushik et al. AAC. 2019 = reference 20 in the manuscript). Therefore, as discussed in our manuscript (lines 324 to 331),a “possible explanation why the 20 mg/kg was less effective when administered as a LA injection rather than orally is that the doses tested in our experiment were too low for LA-IM BDQ. Indeed, in the studies evaluating the BD-LA IM in tuberculosis higher doses were used and it was shown that BDQ LA-IM 160 mg/kg generated a Cmax equivalent to that of 30 mg/kg oral BDQ. In support of this hypothesis is the fact that in the present study when comparing 20 mg/kg LA-IM BDQ to 2 mg/kg oral BDQ at equivalent dosing, the injectable form was more active than the oral form. Consequently BDQ LA-IM should be further tested at higher dosing against leprosy.”*

C2: Since the lower limit of detection is around 4.24 log10, it may not be accurate to compare between groups using log reduction values that assume that all mice without detectable AFB had zero AFB when the AFB could really lie anywhere in the range from zero to 4 log10. Suggest to avoid referring to log reductions that involve groups with AFB below the limit of detection.

***Answer****: see response to a similar comment from reviewer 2 here above.*

C3: For readers unfamiliar with the proportional bactericidal model, it may be difficult to understand how the value for “% viable M. leprae” in Table 2 was derived. Please describe in more detail. For example, the authors could illustrate how the value of 2.753% was obtained for the untreated control in the results section.

***Answer****: we understand reviewer’s comment, however, the statistical method behind the calculations is complex and not easily explained in a few sentences. It is why all the papers published since this method was described refer to it without reexplaining it. However, we propose to add the following explanations in the result section (lines 255 to 261): “the proportion of viable M. leprae surviving the treatment could be calculated by estimating the “most probable number” of viable organisms. However, the estimation of the MPN is based on the assumption that the organisms are distributed randomly in an inoculum; in the case of M. leprae, this assumption is probable untenable, therefore, the preferred alternative is to calculate the “median infectious dose (ID50)”, i.e. the number of organisms required to infect 50% of the mice as allowed by the Spearman-Kärber method (it requires that the titration carried out over a range of 100% to 0%). In immunocompetent mice, if the largest inoculum is 5X10^3^ M. leprae per foot-pad, a proportion of viable M. leprae as small as 0.00006 may be measured, then it is possible to calculate the proportion of viable M. leprae killed by the treatment by comparing the proportions of viables in tested and control mice”.*

Reviewer #3: The results section is well done.

***Answer****: we thank the reviewer for his positive comment.*

--------------------

<b>Conclusions

-Are the conclusions supported by the data presented?

-Are the limitations of analysis clearly described?

-Do the authors discuss how these data can be helpful to advance our understanding of the topic under study?

- Is public health relevance addressed?

Reviewer #1: Clear and justified.

***Answer****: we thank the reviewer for his positive comment.*

Reviewer #2:

C1: The interpretation of the results of treatment with BDQ and CFZ in combination requires some revision. Since the efficacy of CFZ monotherapy at the dose tested was far greater than that of BDQ monotherapy at the doses tested in the combination, the proper conclusion is not that CFZ adds to the efficacy of BDQ but rather than BDQ does not add significantly to CFZ at the doses tested. Further work to identify a BDQ dose that increases the efficacy of the combination when added to CFZ appears warranted.

***Answer****: we agree with Reviewer≠2 as the BDQ dosages tested in combination with CFZ were lower which were less active than the BDQ dosages of 25 and 3.3 mg/kg. So, we agree to change the text as suggested by the Reviewer≠2.*

Reviewer #3:

C1: The conclusion is drafted with good discussion and references to highlight findings, usage implications and future research.

***Answer****: we thank the reviewer for his positive comment.*

C2: The first sentence of the discussion should be rephrased as duration of treatment is not the only challenge. Consider: "The current length of leprosy treatment remains a challenge." It may also be beneficial to cite Indian reports on leprosy treatment default or, more simply, the International Textbook of Leprosy's chapter on "Treatment of Leprosy" (https://internationaltextbookofleprosy.org/chapter/treatment). See the paragraph with default rates in some Indian studies ranging up to 34% in MB cases.

***Answer****: the suggested modification and reference to add have been made.*

--------------------

Editorial and Data Presentation Modifications?

Use this section for editorial suggestions as well as relatively minor modifications of existing data that would enhance clarity. If the only modifications needed are minor and/or editorial, you may wish to recommend “Minor Revision” or “Accept”.

Reviewer #1:

C1: Line 99: 'deserve' should be 'deserves'

***Answer****: requested change has been made.*

C2: Line 133: 'grinded' should be 'ground'

***Answer****: “ground” has a different signification than “grinded”. Since the footpads need to be grinded we would like to keep that word in the sentence. However, the sentence has been slightly modified to be clearer.*

Reviewer #2:

C1: Line 99: “Rv0678” not “Rv6708”

***Answer****: requested change has been made.*

C2: Line 100: consider adding a more detailed description of the gene product beyond “inhibitor of efflux pumps” and provide the pseudogene name that is being referred to here, if possible

***Answer****: a more detailed description of gene product beyond “inhibitor of efflux pumps” was provided (line 100 to 103): “mmpR (Rv0678) gene encoding the Mycobacterium tuberculosis MmpS5–MmpL5 efflux pomp repressor”*

C3: Line 101: does the same statement apply for clofazimine?

***Answer****: it is likely but it has never been evaluated as far as we know.*

C4: Line 112: delete “standard of”?

***Answer****: requested change has been made.*

C5: Line 114: suggest to replace “the oral BDQ” with “BDQ administered orally” or “orally administered BDQ”

***Answer****: requested change has been made.*

C6: Line 133: “was” not “were”

***Answer****: requested change has been made.*

C7: Lines 134 and 137: Hanks’ balanced salt solution

***Answer****: requested change has been made.*

C8: Line 141: please confirm whether the BDQ-LA IM is the same formulation that was studied in references 21-23 and confirm the concentration of BDQ in the formulation. If it is not the same, please describe the differences.

**Answer:** yes, the BDQ-LA IM formulation used in the current study is the same that was used in references 21-23.

C9: Line 146: “combined with” not “combined to”

***Answer****: requested change has been made.*

C10: Lines 148-9 and elsewhere: suggest to use either “the Shepard method” or “Shepard’s method” but not “the Shepard’s method”

***Answer****: requested change has been made.*

C11: Line 149: and elsewhere, if 5.103 is the same as 5x103, the latter may be a more conventional way to represent it

***Answer****: requested changes have been made overall the manuscript.*

C12: Line 174: delete “were” OR change “in” to “if”?

***Answer****: the requested change has been made.*

C13: Line 186-7: for readers unfamiliar with the model, it may be unclear what is meant by “50% infectious dose”. Perhaps the sentence would be clearer if rephrased with added detail as “The proportion of viable M. leprae after treatment was determined from the infectious dose required to show multiplication in 50% of the inoculated mice” or something similar.

***Answer****: the requested change has been made.*

C14: Line 197: “…0.26 log10 AFB per footpad”

***Answer****: the requested change has been made.*

C15: Lines 208-10: since the activity of CFZ was clearly greater than that of low-dose BDQ, it would be more appropriate here to ask if addition of BDQ increased the activity of CFZ, not the other way around. The answer appears to be “probably not”.

***Answer:*** *see also the first comment of the reviewer #2 above in the “Conclusion section” concerning the same point (comment Reviewer #2 C1 “Conclusion section”). We agree that the question is that low doses of BDQ can improve the activity of CFZ and the answer is likely yes despite the slight improvement both regarding the number of AFB negative mice and the reduction in AFB counts were not significant. The manuscript has been modified as answered above to the related previous comment.*

C16: Line 214: suggest to replace “association” with “addition” or rephrase as “the combination of CFZ with BDQ”

***Answer****: the requested change has been made.*

C17: Line 215: suggest to rephrase “during 24 weeks one month…” as “for 24 weeks beginning one month…”

***Answer****: the requested change has been made.*

C18: Line 224: perhaps it would be clearer to rephrase the first sentence as “From results in the untreated group, the proportion of viable M. leprae was estimated to be 2.75% of the total number of AFB inoculated”?

***Answer****: the requested change has been made.*

C19: Line 251: when saying “few new molecules were synthesized”, please describe the therapeutic area you are referring to. For example, anti-infectives or anti-tuberculosis drugs?

***Answer****: the requested change has been made.*

C20: Line 258: consider replacing “similar to” with “indistinguishable from”. It is a subtle difference but would better reflect the fact that differences that all footpad CFU counts were below the limit of detection and any dose-related differences that may have been apparent with shorter durations of treatment were not assessable

***Answer****: the requested change has been made.*

C21: Line 296: would specify that BDQ-LA was active in a mouse model of latent TB

***Answer****: the requested change has been made.*

C22: Line 309: given the preceding discussion, should it be specified that this MED is for immunocompromised mice?

***Answer:*** *we have the feeling that this precision will make the discussion more difficult to read; therefore, we would not add this specification there again.*

C23: Table 1: for the p value column, it would be helpful to indicate that this p value pertains to comparisons made on the basis of AFB/fp and not the proportion of footpads that are positive

***Answer****: see answer to C3 question from reviewer 2 in the first section.*

C24: Table 2: in the 5.102 column, the BDQ-LA IM 20 mg/kg SD group should have “3/10” not “3/0”

***Answer****: the requested change has been made.*

C25: Table 2: there appears to be an error in the RIF SD group with either the % viable M. leprae value of 0.432 or the % killed value of 90.01. Please check the values and confirm the accuracy of the statistical result.

*Answer: unfortunately, there was a mistake in the table which was corrected in the revised version.*

Reviewer #3:

C1: Some sentences in the introduction should be rephrased or simplified for clarification. For example, "The next goal which remains to be achieved is to develop a strategy focusing on zero leprosy by the end of 2030." The WHO strategy for 2021-2030 is cited (3) and was developed during 2019-2020. The authors perhaps meant "The WHO global strategy targets zero leprosy by 2030. This goal has been hindered by (i) sharp reduction of leprosy case detection during 2020-2021 due to the pandemic (see other suggested citations below), (ii) ... this next part of the sentence becomes convoluted and could be a separate sentence on its own.

WHO Weekly Epidemiological Record - every September issue includes an annual report on the previous year of global leprosy diagnoses. Therefore, 2021 data is reported in the 2022 edition - which adds more info relevant to your point.

2021: https://reliefweb.int/report/world/weekly-epidemiological-record-wer-9-september-2022-vol-97-no-36-2022-pp-429-452-enfr

***Answer****: the requested changes and reference to add have been made.*

C2: Line 85: concomitant drug interactions can be another reason

***Answer****: the requested change has been made.*

C3: Line 111: does BDQ have the longest half-life of current MDT and recognized second line treatment drugs for leprosy? If so, that could be beneficial to state.

**Answer:** yes, BDQ has the longest half-life of all the drugs used to treat leprosy (MDT and second line drugs such as ofloxacin, minocycline and clarithromycin).

C4: The sentence on lines 283-284 should be rephrased for clarity. Consider: When combined with low doses of BDQ that were ineffective alone, CFZ enabled reduced numbers of AFB positive footpads.

***Answer****: the requested change has been made taking also into account above C1 comment from reviewer 2 in the “Conclusions” section (lines 304 to 306).*

--------------------

Summary and General Comments

Use this section to provide overall comments, discuss strengths/weaknesses of the study, novelty, significance, general execution and scholarship. You may also include additional comments for the author, including concerns about dual publication, research ethics, or publication ethics. If requesting major revision, please articulate the new experiments that are needed.

Reviewer #1: This is a straightforward paper with a message of interest to anyone involved in treating leprosy.

***Answer****: we thank the reviewer for his positive comment.*

Reviewer #2:

In this manuscript the authors describe two experiments to evaluate the activity of bedaquiline (BDQ) in mouse footpad infection models of leprosy. The first experiment evaluated a wide range of daily oral bedaquiline doses administered for 24 weeks beginning 1 month after infection in immunocompromised nude mice to determine the minimal effective dose (MED) of BDQ and included additional arms with CFZ alone and in combination with low dose BDQ. The second experiment evaluated two dose levels of BDQ (2 and 20 mg/kg) administered either orally or as a long-acting (LA) formulation, and either as a single dose (SD) or as 3 doses (3D) spaced 4 weeks apart, beginning immediately after infection in immunocompetent mice. In the first experiment, BDQ doses of 0.1, 0.33 and 1 mg/kg/d had modest but statistically significant effects on the AFB burden compared to no treatment, while doses of 3.3 and 25 mg/kg reduced the burden to levels below the limit of detection. Hence,

the MED was determined to be 3.3 mg/kg. CFZ alone was also highly active and it was not apparent that adding BDQ at doses up to 0.33 mg/kg/d increased its activity. In the second experiment, BDQ showed dose-dependent and duration-dependent activity. At 2 mg/kg, results in SD arms were not statistically significantly different from no treatment, while 3D arms killed ~1 log10. Oral and LA forms had similar activity at this dose level. However, while both oral and LAI forms had significant killing effects at the 20 mg/kg dose level, the oral formulation was more active than the LA formulation at this dose level. The authors conclude that BDQ may be effective against leprosy at doses lower than those used for TB, that addition of CFZ may improve the efficacy of BDQ, and that LA BDQ formulations should be tested at higher doses in future experiments.

The experiments yield novel and timely information since initial efforts are underway to evaluate the activity of daily oral BDQ in leprosy patients. However, the potential impact of this study on future preclinical and clinical development efforts is more difficult to judge. Some of the comments and questions provided are intended to help the reader to better understand the authors’ perspectives.

***Answer****: we thank the reviewer for his very helpful comments which helped us a lot to improve our manuscript.*

Reviewer #3: The novel information reported in this manuscript is critically important to improving best care options for leprosy cases. The work was done well in both competent and immunocompetent mouse models to best depict drug combination and regimen impact on M.leprae with clear connection to feasibility in patient care. The immunological and drug dynamics were well explained and referenced. There are only a few minor edits that could potentially improve clarity. Overall, it is a superb paper.

***Answer****: we thank the reviewer for his very positive comment.*
