# Supplementary material for: Minimal effective dose of bedaquiline administered orally and activity of a long acting formulation of bedaquiline in the murine model of leprosy
Source: PLoS Negl Trop Dis. 2023 Nov 27;17(11):e0011379. doi: 10.1371/journal.pntd.0011379 (PMC10703217; doi:10.1371/journal.pntd.0011379)
Supplement: Attachment — Submitted filename: PNTD_D_23_00593__Response to Reviewers_FINAL.docx [file pntd.0011379.s002.docx]

**Answers to reviewers**

Reviewer #2:

C1: Line 23: suggest to replace “adding” with “combining”

***Answer****: requested change has been made.*

C2: Lines 100-3: wouldn’t an inactivated mmpR gene be more likely to contribute to a lower potency of bedaquiline against M. leprae due to loss of the efflux repressor?

***Answer****: The sentence was confusing because M. leprae loss the entire efflux pump, not only the repressor; therefore the sentence was rephrased in the manuscript and a reference was added. Overall, M. leprae which is often considered to possess a “minimal” mycobacterial genome as a result of reductive evolution, possess also a “minimal” set of five intact mmpL genes (mmpL3/4/7/10/11) as well as a similarly split mmpL13a/b locus; the mmpS5-L5 genes being absent (see review, i.e. reference 20).*

C3: Line 141: consider to replace “compare” with “represent”

***Answer****: requested change has been made.*

C4: Line 216: suggest to replace “different” with “significant“

***Answer****: requested change has been made.*

C5: Line 228: suggest to replace “number” with “proportion”

***Answer****: requested change has been made.*

C6: Line 257: replace “probable” with “probably”

***Answer****: requested change has been made.*

C7: Line 258: “…titration be carried out…”

***Answer****: requested change has been made.*

C8: Line 259: replace “foot-pad” with “footpad”

***Answer****: requested change has been made.*

C9: Table 1: it is not clear what is meant by “median value on positive footpads” at the top of the 3rd column.

***Answer****: we changed “median value” by “standard deviation”.*

Summary and General Comments

Use this section to provide overall comments, discuss strengths/weaknesses of the study, novelty, significance, general execution and scholarship. You may also include additional comments for the author, including concerns about dual publication, research ethics, or publication ethics. If requesting major revision, please articulate the new experiments that are needed.

Reviewer #2:

Reviewer #2: The authors' responses and revisions have adequately addressed the issues raised in the prior reviews. A few minor editorial comments are provided above.

***Answer****: we thank the reviewer for his positive comment.*
